# Environmental determinants of COVID-19 transmission across a wide climatic gradient in Chile

**DOI:** 10.1038/s41598-021-89213-4

**Published:** 2021-05-10

**Authors:** Francisco Correa-Araneda, Alfredo Ulloa-Yáñez, Daniela Núñez, Luz Boyero, Alan M. Tonin, Aydeé Cornejo, Mauricio A. Urbina, María Elisa Díaz, Guillermo Figueroa-Muñoz, Carlos Esse

**Affiliations:** 1 Unidad de Cambio Climático y Medio Ambiente, Instituto Iberoamericano de Desarrollo Sostenible, Universidad Autónoma de Chile, Temuco, Chile; 2 Facultad de Ciencias de la Salud, Universidad Autónoma de Chile, Temuco, Chile; 3 Programa de Doctorado en Ciencias Mención en Ecología y Biología Evolutiva, Facultad de Ciencias, Universidad de Chile, Santiago, Chile; 4 Department of Plant Biology and Ecology, Faculty of Science and Technology, University of the Basque Country (UPV/EHU), 48940 Leioa, Spain; 5 IKERBASQUE, Basque Foundation for Science, Bilbao, Spain; 6 Aquariparia/Limnology Lab, Department of Ecology, IB, University of Brasília (UnB), Brasília, Brazil; 7 Aquatic Ecology and Ecotoxicology Laboratory. Zoological Collection Eustorgio Mendez, Gorgas Memorial Institute for Health Studies (COZEM-ICGES), Ave. Justo Arosemena and Calle 35, 0816-02593 Panama City, Panama; 8 Departmento de Zoología, Facultad de Ciencias Naturales y Oceanográficas, Universidad de Concepción, P.O. Box 160-C, Concepción, Chile; 9 Instituto Milenio de Oceanografía (IMO), Universidad de Concepción, PO Box 1313, Concepción, Chile; 10 Departamento de Ciencias Ambientales, Facultad de Recursos Naturales, Universidad Católica de Temuco, Temuco, Chile

**Keywords:** Epidemiology, Atmospheric science

## Abstract

Several studies have examined the transmission dynamics of the novel COVID-19 disease in different parts of the world. Some have reported relationships with various environmental variables, suggesting that spread of the disease is enhanced in colder and drier climates. However, evidence is still scarce and mostly limited to a few countries, particularly from Asia. We examined the potential role of multiple environmental variables in COVID-19 infection rate [measured as mean relative infection rate = (number of infected inhabitants per week / total population) × 100.000) from February 23 to August 16, 2020 across 360 cities of Chile. Chile has a large climatic gradient (≈ 40º of latitude, ≈ 4000 m of altitude and 5 climatic zones, from desert to tundra), but all cities share their social behaviour patterns and regulations. Our results indicated that COVID-19 transmission in Chile was mostly related to three main climatic factors (minimum temperature, atmospheric pressure and relative humidity). Transmission was greater in colder and drier cities and when atmospheric pressure was lower. The results of this study support some previous findings about the main climatic determinants of COVID-19 transmission, which may be useful for decision-making and management of the disease.

## Introduction

On the last day of 2019, a viral outbreak of unknown origin was detected in a seafood market in Wuhan City, Hubei Province, China^1^. This virus, later named *severe acute respiratory syndrome* coronavirus 2 (SARS-CoV-2; Coronaviridae) and responsible for the clinical disease known as COVID-19, has now spread around the globe and declared a pandemic by the World Health Organization (WHO) on March 12, 2020. To date (March 1, 2021), it has been detected in 219 countries, with about > 115 million people infected and > 2.5 millions deaths worldwide^2^. The global scale and severity of the impacts of this disease, despite being at an intermediate stage of development, is unprecedented, and studies have suggested that it might take more than a decade for the world to recover from its effects, both socially and economically^3^.

Upon appearance of COVID-19, several studies have examined the transmission dynamics of this disease^4^. While some have documented the route of transmission through human-to-human contact, others have examined the role that some environmental factors may play in facilitating the rate of spread of the disease through the analysis of temporal and spatial relationships of these factors with COVID-19 transmission rate. Most of these studies have reported a negative relationship between transmission rate and several proxies of temperature and humidity, suggesting that the disease spread is enhanced in colder and drier climates^5–9^.Other environmental variables have received less attention and results have been inconclusive or differed among countries. For example, one study reported an inverse relationship between COVID-19 transmission and wind speed in Iran^10^, while global studies have found no significant association between both variables^11,12^. A negative relationship of the disease transmission with solar radiation has been reported in Iran^10^, and a positive relationship with the concentration of atmospheric pollutants was found in China^13^. Overall, and despite the rapid response of the scientific community to understand the transmission of COVID-19, the role that environmental variables play in the disease dynamics remains an open question that requires further evidence across the world.

The goal of this study was to examine the potential role of multiple environmental variables in COVID-19 transmission rates and patterns in Chile. Environmental variation within Chile is unique due to the particular geography of this country, which includes an altitudinal range of ≈ 7000 m from the sea level to the top of Aconcagua mountain, and a ≈ 40º latitudinal gradient that covers 6 climatic zones, including desert, semiarid, mediterranean, marine west coast, tundra and ice sheet. At the same time, population across the country shares a common social behaviour, and regulations are established by a single national authority, allowing the evaluation of environmental variables under relatively consistent socio-economic conditions^14^. We thus aim to provide information about COVID-19 transmission across a wide range of environmental variation within a single country that may help understanding the dynamics of this disease.

## Results

### Summary of study area characteristics and COVID-19 transmission data

**Figure 1:**
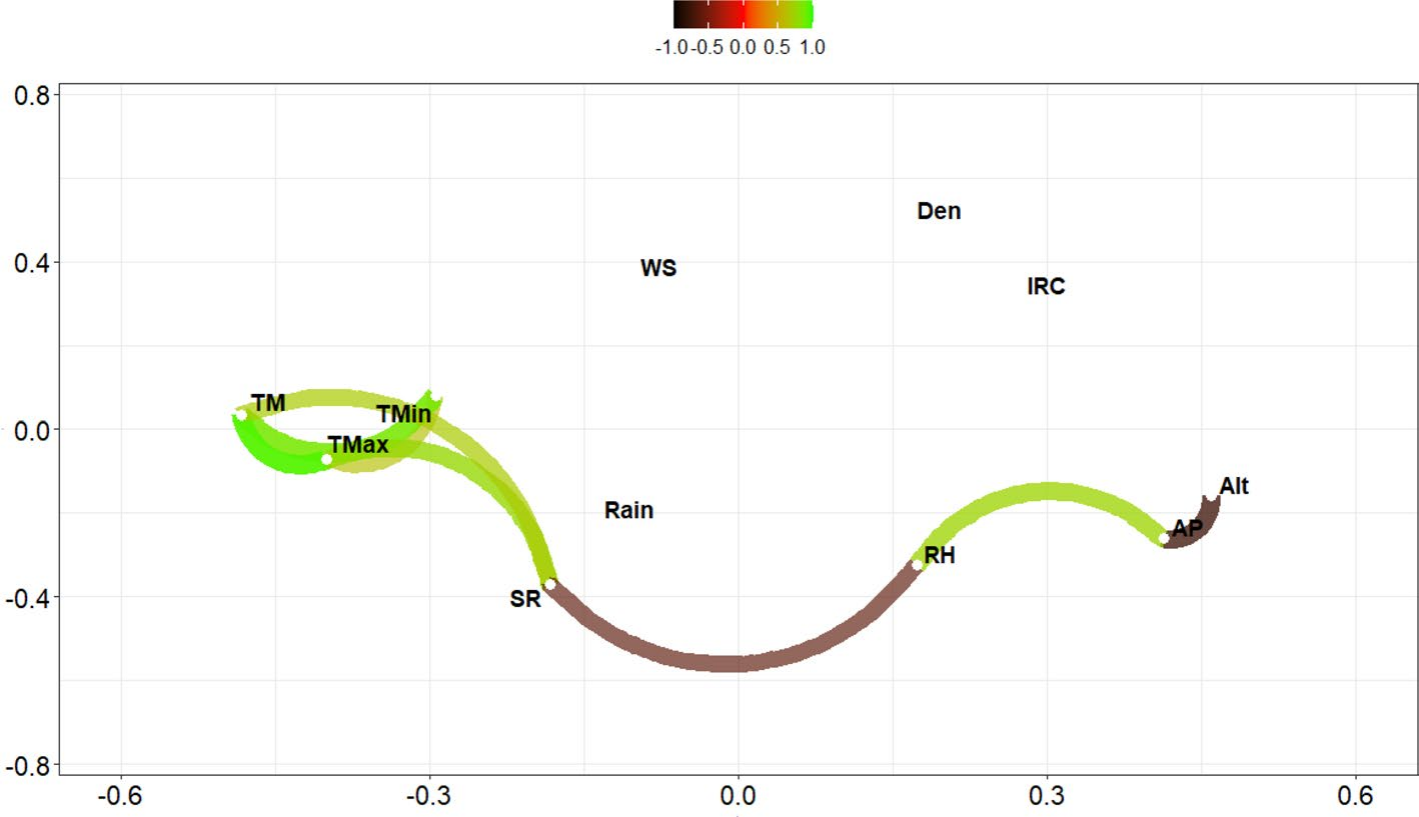
Correlations plot of environmental database COVID-19. Green paths indicate positive correlations, brown paths negative correlations. TM= Average atmospheric temperature; TMax = Maximum ﻿atmospheric temperature; TMin = Minimum atmospheric temperature; RH = Relative humidity; Rain = Accumulated precipitation; AP = Atmospheric pressure; SR = Ultraviolet solar radiation; WS = Wind speed; Alt = Elevation; Den = Population density; IRC = Infection rate.

Mean temperature varied between − 11.99 °C (polar macroclimate) and 23.69 °C (semi-arid); relative humidity, between 2.56% (desert) and 96.83% (polar); atmospheric pressure, between 600.29 mbar (semi-arid) and 1062.71 mbar (marine south west); and wind speed, between 0 km h^−1^ (mediterranean) and 64.01 km h^−1^ (polar) (Table 1). Absolute population size ranged from 137 inhabitants (Antarctica) to 645,909 inhabitants (Puente Alto, mediterranean) (Fig. 1; see Supplementary Table S1 online). The first registered COVID-19 infection case in Chile occurred on February 23, 2020; to the data collection date (August 16, 2020), more than 387,000 infected inhabitants and 10,500 deaths have been reported. The mean infection rate ranged from 0 (several cities) to 4444 in General Lagos (desert) in week 32, and the number of weekly infections from 0 (several cities) to 2549 (Puente Alto) (see Supplementary Table S1 online).

**Table 1:** Mean values (standard error) of the variables recorded between February 23 to August 16, 2020 in relation to climates.

	Desert (n = 650)	Semiarid (n = 350)	Mediterranean (n = 5925)	Marine South West (n = 1225)	Tundra (n = 500)
Altitude (m a.s.l.)	1298 (1300.2)	439 (336)	285.2 (247.7)	88.0 (92.5)	139.2 (152.5)
Population size (n° inhabitants)	60,309.2 (104,458.6)	57,790.3 (85,457.7)	64,774.9 (94,591.6)	29,500.1 (48,398.9)	14,203 (32,593.9)
Pop. density (inhabitants km^−2^)	17.62 (45.21)	29.55 (52.94)	1482.2 (3779.1)	29.0 (41.1)	1.1 (2.4)
Maximum temperature (°C)	20.2 (4.4)	19.6 (4.7)	18.0 (6.0)	14.2 (4.6)	8.9 (5.8)
Minimum temperature (°C)	7.0 (8.3)	8.9 (4.2)	6.2 (3.4)	5.7 (2.7)	2.0 (4.4)
Mean temperature (°C)	13.6 (5.4)	14.3 (4.0)	12.1 (4.3)	9.9 (3.3)	5.5 (4.9)
Relative humidity (%)	53.2 (24.07)	56.0 (20.7)	71.9 (11.8)	81.9 (6.9)	77.6 (9.4)
Accumulated precipitation (mm)	0.4 (1.6)	0.3 (1.1)	2.6 (4.1)	8.5 (12.8)	2.4 (4.3)
Atmospheric pressure (mbar)	857.4 (150.1)	890.5 (127.3)	987.7 (40.6)	1001 (21.8)	994.5 (21.7)
Solar radiation (Mj m^−2^)	17.75 (4.8)	14.1 (4.9)	10.8 (5.9)	7.9 (4.9)	5.6 (3.7)
Wind speed (km h^−1^)	5.95 (3.55)	6.7 (4.0)	4.8 (3.1)	5.5 (3.5)	10.2 (8.1)
MP2.5 (µg m^−3^)	10.6 (3.9)	11.6 (2.9)	27.6 (17.5)	42.8 (25.8)	19.8 (17.9)
MP10 (µg m^−3^)	36.41 (12.3)	34.3 (11.1)	50.3 (23.2)	49.3 (26.2)	N.D.
Mean relative infection rate	122.4 (273.5)	37.8 (63.6)	53.9 (85.5)	13.6 (28.9)	9.0 (35.7)

*N.D.* no data.

### Relationship between COVID-19 and predictive variables matrices

The major statistical associations between the variables were registered without the time lags analyzed (0 days). The correlation analysis showed a strong relationship between the average, maximum and minimum temperatures, as well as between altitude and relative humidity, atmospheric pressure and solar radiation. There was no correlation between population density, wind speed and IR, and low correlation between precipitation and all other variables (Fig. 1). According to multicollinearity analysis, the variables of greatest importance were minimum and mean temperature (variance inflation factor, VIF > 10) and the least important was maximum temperature (VIF < 10). Given the above, the reduced the data matrix excluded mean and maximum temperature and altitude as predictor variables.

**Figure 2:**
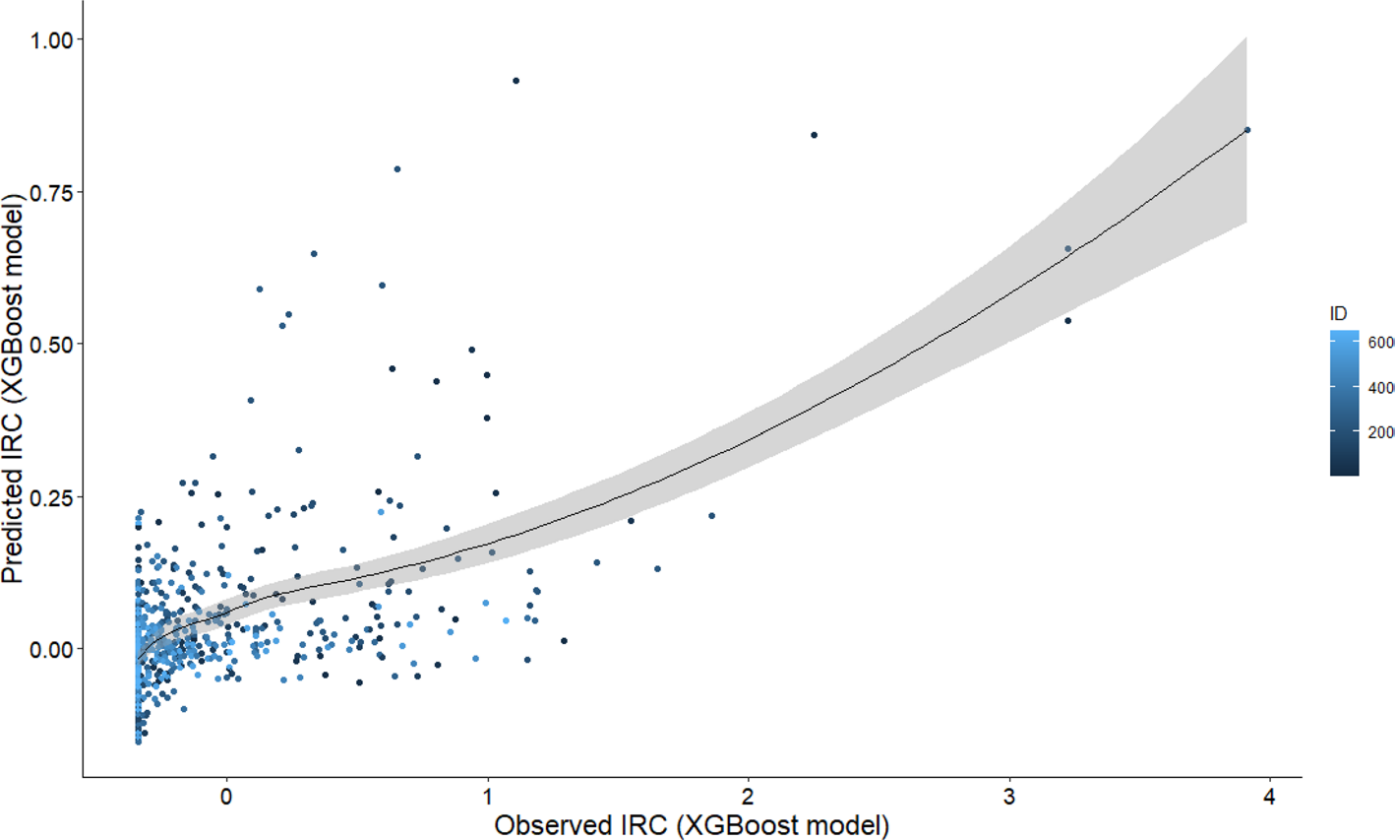
Scatter plot of predicted IRC versus observed values using the XGBoost method.

**Figure 3:**
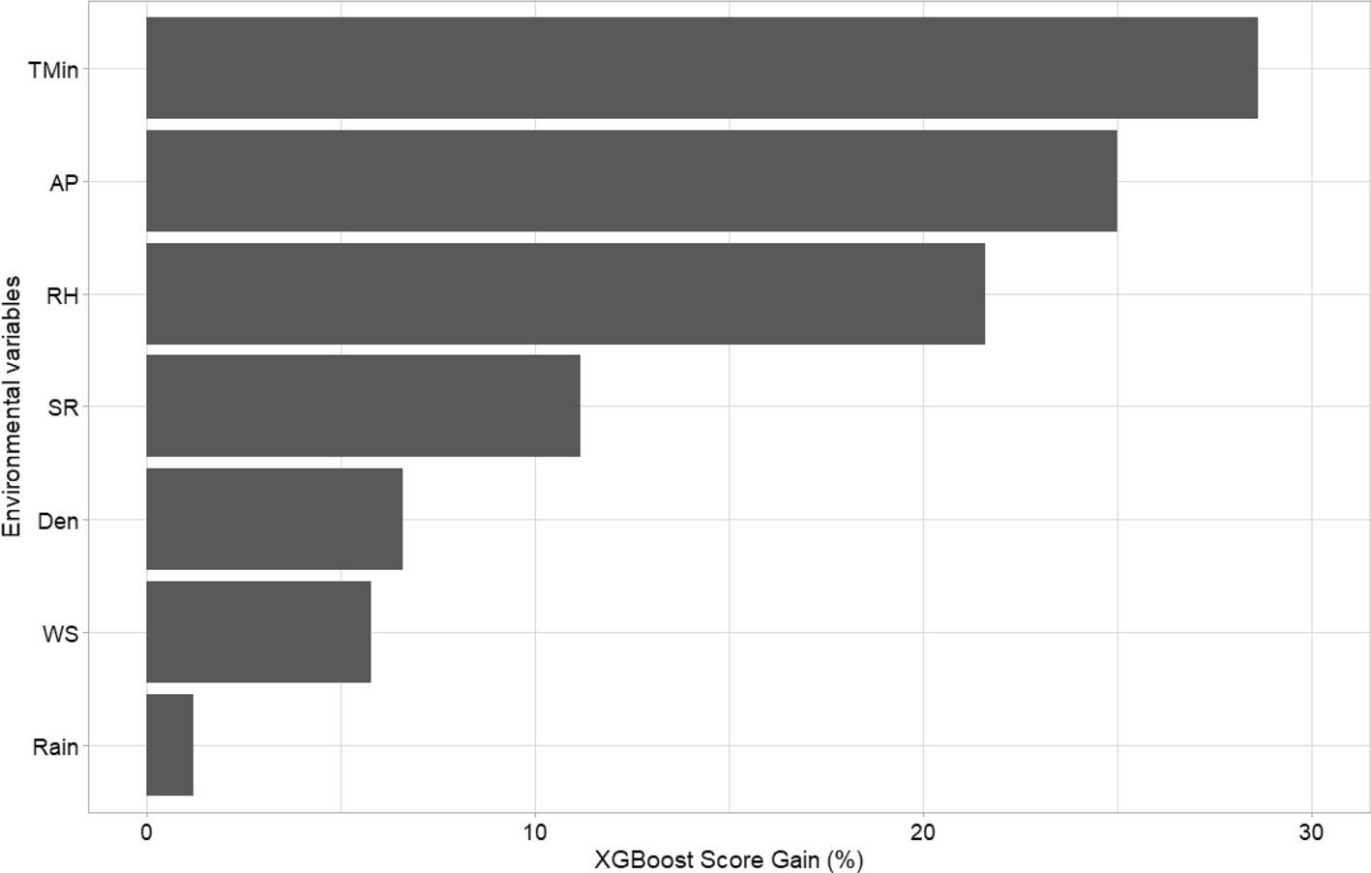
Top 7 most important variables based on the Gain Score (impurity) metric. TMin = Minimum atmospheric temperature; AP = Atmospheric pressure; RH = Relative humidity; SR = Ultraviolet solar radiation; Den = Population density; WS = Wind speed; Rain = Accumulated precipitation.

The training model was adjusted using the database with 3368 observations and 7 predictive variables. The hyperparameter selection allowed improving the predictions. The final tuning values used for the model were n_estimators (number of iterations in training) = 56, max_depth (maximum depth of the tree) = 4, eta (model learning rate) = 0.03, gamma (Minimal loss reduction required to perform an additional partition on a leaf node of the tree) = 0, colsample_bytree (the last parameter that we need to config) = 0.5, min_child_weight (sum of sample weight of the smallest leaf nodes to prevent overfitting) = 1 and subsample (sampling rate of all training samples) = 1 (Table 2). The predictions corresponding to week 0 showed a lowest error with 56 iterations (Table 3). The scatter plot of predicted mean relative infection (IR) versus observed values using the final model is illustrated in Fig. 2. The scatterplot considering all parameters demonstrate an acceptable prediction of IR with a R^2^ = 0.32 (R = 0.57). The Gain Score showed that the most important variables were minimum temperature (Tmin), atmospheric pressure (AP) and relative humidity (RH) (Fig. 3). All these final selected variables showed a highly significant negative relationship with the infection rate (p < 0.0001; Tmin r = − 0.25, AP r = − 0.23, RH r = − 0.21).

**Table 2:** Extreme Gradient Boosting regression modeling hyperparameters from the grid search.

Parameter	Description	Range	Optimum value
n_estimators	Number of iterations in training	10–1000	56
Eta	Model learning rate	0.02–0.05	0.03
max_depth	Maximum depth of the tree	4–8	4
Gamma	Minimal loss reduction required to perform an additional partition on a leaf node of the tree	0	0
colsample_bytree	The last parameter that we need to config	0.5–0.9	0.5
min_child_weight	Sum of sample weight of the smallest leaf nodes to prevent overfitting	1–2	1
subsample	Sampling rate of all training samples	1–3	1

Optimum value = hyperparameter selected for the improved model (tuning).

**Table 3:** Root mean square error (RMSE) obtained for each database from the adjusted predictive model.

Week/days	Best iteration	RMSE-test	RMSE-prediction 1	RMSE-prediction 2
0/0	56	0.8239	0.5345	0.5238
1/7	34	0.7017	1.4153	1.4449
2/14	14	0.843	0.7641	0.8434

RMSE-test = error of the model fitted by default; RMSE-prediction 1 = error of prediction without hyperparameters; RMSE-prediction 2 = error with use of hyperparameters.

## Discussion

Our results demonstrate that COVID-19 infection rate in Chile to date has been linked to 3 main environmental variables: minimum temperature, atmospheric pressure and relative humidity. Firstly, we found a negative relationship between infection rate and minimum temperature. Other studies have reported a similar, negative relationship between air temperature and the transmission of COVID-19^15–18^ and other respiratory diseases such as SARS^19^. However, a positive correlation with average and minimum temperature has been reported in Singapore, especially in the initial phase of transmission^20^. Others have found an indirect, positive effect of average temperature on the spread of the SARS-CoV-2 virus due to enhanced people’s mobility at higher temperatures^21^. These findings are particularly concerning at present in the southern hemisphere, which is entering winter and therefore lower temperatures are expected in the coming months, which could drive an upsurge of the disease.

Atmospheric pressure was the second relevant variable and it was negatively related with the spread of the SARS-CoV-2 virus. The link between atmospheric pressure and the spread of the SARS-CoV-2 virus has been studied in several countries^20–26^, since atmospheric pressure is responsible for air movement (wind), cloud formation, precipitation and humidity. Therefore, this variable has strong influence on climatic variation, generating favourable conditions for the virus spread in some cases (drought and light wind) but not in others (high humidity and strong wind). Others have provided evidence for a direct link between atmospheric pressure and the virus spread, indicating that the unusual persistence of an anticyclonic atmospheric situation (i.e., abnormally strong positive phase of the North Atlantic and Arctic oscillation) in southwestern Europe, centered in Spain and Italy during February 2020, generated conditions of drought and light wind that could have favored the faster spread of the virus compared to other European countries^22^. This is reinforced by the positive correlation found between atmospheric pressure and the frequency of COVID-19 cases in Mozambique^25^, and with several spread parameters (infection rate, effective reproduction number and compound growth rate) in 487 cities in the United States^23^. Such positive relationship could be related to an increase in fog associated to high pressure, which increases the humidity of the air and surfaces. However, other studies have found an inverse link between atmospheric pressure and the spread of the SARS-CoV-2 virus in Singapore and China^20,26^, which could be explained by the fact that high pressures can limit suspension time of viral particles in the environment^26^. Indirectly, atmospheric pressure could also reduce the virus spread by limiting people's mobility^21^. Overall, there is no consensus on the link between atmospheric pressure and the spread of the SARS-CoV-2 virus since there is evidence that describes both direct and inverse correlations, even both within the same country (e.g., Italy)^24^.

The negative relationship that we observed between relative infection rate and relative humidity is consistent with former evidence that high relative humidity reduces the COVID-19 viability^27,28^ and transmission rates^7,29^. Similarly, high relative humidity has been reported to reduce the survival of the influenza virus^30^ and the incidence of this disease^8^. Environmental humidity can affect viral transmission through its interaction with respiratory droplets, which act as virus containers and can remain longer in dry air^31,32^. Additionally, high relative humidity leads to inactivation of the viral lipid membrane, and consequently a decrease in the virus stability and transmission^33,34^. However, a study found a direct link between average relative humidity and the SARS-CoV-2 basic reproductive ratio in China^26^. Again, relative humidity can indirectly contribute to the spread of the SARS-CoV-2 virus due to its influence on people’s mobility^21^.

In conclusion, our study shows that climate plays a key role in the transmission of COVID-19 in Chile, a country that comprises a particularly high variation of environmental conditions. Importantly, it is highly likely that climatic conditions expected for the coming months in the southern hemisphere (i.e., lower temperature, humidity and atmospheric pressure) can favour a higher disease transmission speed. Our study and others providing information about how climatic factors can influence the spread of the disease may serve as the basis for predictive models of COVID-19 transmission through space and time, which will be highly relevant to decision-making and management of the disease.

## Materials and methods

### Study area

**Figure 4:**
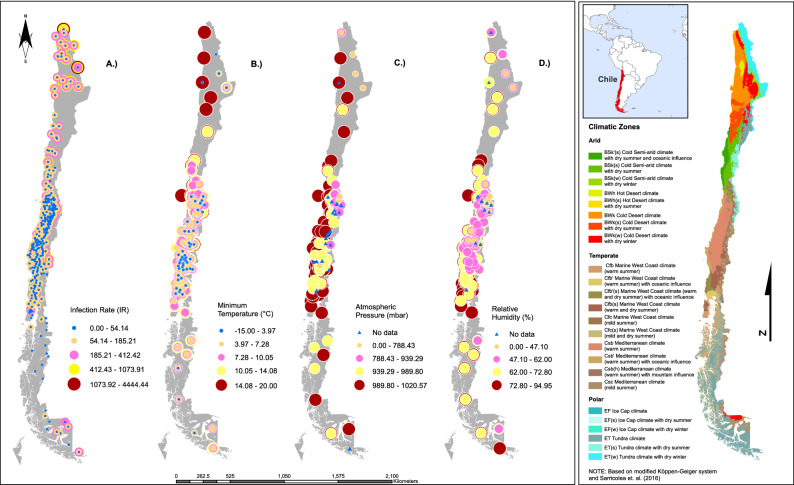
Map of Chilean distribution of (**A**) infection rate, (**B**) minimum temperature (°C), (**C**) atmospheric pressure (kPa), and (**D**) relative humidity (%).

We examined data from 360 ‘communes’ or cities in Chile, which are distributed across 4200 km from north to south. Latitudes of our study area range from 17° S (Arica) to 56° S of latitude (Cabo de Hornos), and altitudes range from 8 m a.s.l. (Pacific Ocean coast) to 3962 m a.s.l. (San Pedro de Atacama, Andean mountain range). The study area covers the following five climatic zones: (i) desert (17°30′–26°00′ S), (ii) semiarid (26°00′–32°00′), (iii) mediterranean (32°00′–39°00′), (iv) marine west coast (39°00′–44°00′ S) and (v) tundra (44°00–56°00′ S); the only climatic zone excluded from the study was the ice sheet (located in the highest areas of the Andes mountain range) because of the absence of human population. In terms of macroclimates, ca. 41% of the country is temperate, 31% arid and the remaining 28% has a polar climate^36^ (Fig. 4).

Chilean population is 19.11 million inhabitants, of which 51% are women and 49% men. Life expectancies are 83 (women) and 78 (men) years old; 68.7% of the population is between 15 and 64 years old and 11.9% over 65 years old. The 88% of inhabitants live in urban areas and the estimated international migration rate is 12 per thousand inhabitants. The 13% of the population belongs to indigenous or native groups; 80% Mapuche, 7% Aymara and 4% Diaguita^37^. The population is aging as a result of the decline in fertility and the increased life expectancy^38^.

Chile has 16 administrative regions^35,39^, of which the Metropolitana region concentrates the largest population (7.1 million inhabitants), followed by the Valparaiso region (1.8 million inhabitants). In contrast, the Aysén and Magallanes regions, located in the southern extreme of Chile, have the smallest population (< 200,000 inhabitants). Inhabitants > 65 years old mainly inhabit the areas with mediterranean climate in the cities of Santiago, Valparaíso and Concepción, and correspond to 6.28% of the total employed inhabitants in the country^40^. By 2050 it is projected that total population size reaches 21.6 million (i.e., an increase of 15.3% compared to 2020) under assumptions of birth and immigration surpassing mortality and emigration, with inhabitants > 65 years old predicted to exceed 3 million (25% of the population)^38^.

### COVID-19 transmission data and predictive variables

We characterized the COVID-19 transmission in Chile from February 23 to August 16, 2020, based on mean relative infection rate [IR; (number of infected inhabitants per week/total population) × 100,000) of 360 cities. Data were obtained from official sources of the Government of Chile^41^. We extracted daily climatic data from the databases of 159 meteorological stations in Chile^42^ corresponding to cities with and without presence of COVID-19, for the same period; these data were averaged per week to make them comparable with variables quantifying the disease transmission. The data of the climatic variables recorded every 1 h at meteorological stations were extracted and average weekly expressing them as follows: weekly average, maximum and minimum atmospheric temperature (°C); weekly average relative humidity (%; moisture content (i.e., water vapor) of the atmosphere, expressed as a percentage of the amount of moisture that can be retained by the atmosphere (moisture-holding capacity) at a given temperature and pressure without condensation)^43^, absolute humidity (g m^−3^), accumulated precipitation (mm), atmospheric pressure (mbar), ultraviolet solar radiation (Mj m^−2^) and wind speed (km h^−1^). Additionally, we obtained data for other relevant environmental, demographic and geographic variables, those that were averaged and expressed as follows: air pollutant data, including particulate matter with aerodynamic diameter ≤ 10 μm (PM10) and ≤ 2.5 μm (PM2.5), obtained from a database of 30 air quality stations^44^; and city area (km^−2^), population size (ind), population density (ind km ^−2^), latitude (absolute degrees), longitude (absolute degrees) and altitude (m a.s.l.), obtained from CONAF^45^ and IDE Chile^46^.

### Statistical analyses

In order to analyze time lags in the transmission of the virus, three databases were built using environmental information with different time lags of contagion with respect to the response variable (IR), these being: (a) 0 days, (b) 7 days and (c) 14 days. Each database was subjected to an exploratory analysis, which allowed the identification of missing, influential or out of range data, and elimination of variables that were non-influential and/or highly correlated with others. This allowed reducing dimensionality by eliminating redundant information. For this, a correlation matrix was constructed and a boosting model was fitted using the VIF criterion^47^. For the final variables selection were modeled through extreme gradient boosting (XGBoost^48^). This model consists of a successful machine learning library based on a gradient boosting algorithm proposed by Chen^48^, which sequentially processes the data with a loss or cost function, minimizing the error iteration after iteration and increasing predictive power compared to other sequential tree models. We used VIFs to identify multicollinearity^46^ and data were normalized, which is important for machine-learning estimators. Dataset records were shuffled and split to 80% for the training and 20% for the test.

The model was trained with the original parameters adjusting the depth (max_depth) between 1 and 10^49^. Once the best iteration was identified, we proceeded to predict on the validation set. The model was tuned using hyperparameters (Table 2). We used a grid search on hyperparameters, parallelizing the search, with threefold cross-validation was carried out to find the best model based on, root mean square error (RMSE), R^2^ metrics and mean absolute error (MAE). The *xgboost* and *caret* libraries of the R software^50^ were used for the analyzes. Once the most important variables were selected, a spatial representation of each of them and climates was generated for each city, which was adapted from Sarricolea et al.^36^. For this, the existing spatial coverage in shape format in the national database of the Spatial Data Infrastructure IDE-Chile^46^ was used. For the management and analysis of spatial data, the ArcMap software version 10.8.1 (ESRI Inc., Redlands, California, USA) was used.

## Supplementary Information

Supplementary Table S1.

## Data Availability

The raw data was supplied as a Supplementary Information File.

## References

[1] Sarkodie SA, Owusu PA (2020). Investigating the cases of novel coronavirus disease (COVID-19) in China using dynamic statistical techniques. Heliyon.

[2] WHO. *Coronavirus disease (COVID-19) Pandemic*. https://www.who.int/emergencies/diseases/novel-coronavirus-2019 (2021).

[3] United Nations. *Launch of Global Humanitarian Response Plan for COVID-19*. https://www.un.org/sg/en/content/sg/press-encounter/2020-03-25/launch-of-global-humanitarian-response-plan-for-covid-19 (2021).

[4] Li Q (2020). Early transmission dynamics in Wuhan, China, of novel coronavirus-infected pneumonia. N. Engl. J. Med..

[5] Liu J (2020). Impact of meteorological factors on the COVID-19 transmission: A multi-city study in China. Sci. Total Environ..

[6] Luo, W. *et al.* The role of absolute humidity on transmission rates of the COVID-19 outbreak. *medRxiv *2020.02.12.20022467. 10.1101/2020.02.12.20022467 (2020).

[7] Ma, Y. *et al.* Effects of temperature variation and humidity on the death of COVID-19 in Wuhan, China. *Sci. Total Environ.* 138226. 10.1016/j.scitotenv.2020.138226 (2020). 10.1016/j.scitotenv.2020.138226 PMC7142681 32408453

[8] Park JE (2020). Effects of temperature, humidity, and diurnal temperature range on influenza incidence in a temperate region. Influenza Other Respir. Viruses.

[9] Shi, P. *et al.* The impact of temperature and absolute humidity on the coronavirus disease 2019 (COVID-19) outbreak—Evidence from China. *medRxiv* 2020.03.22.20038919. 10.1101/2020.03.22.20038919 (2020).

[10] Ahmadi, M., Sharifi, A., Dorosti, S., Ghoushchi, S. J. & Ghanbari, N. Investigation of effective climatology parameters on COVID-19 outbreak in Iran. *Sci. Total Environ.* 138705. 10.1016/j.scitotenv.2020.138705 (2020). 10.1016/j.scitotenv.2020.138705 PMC7162759 32361432

[11] Chen, B. *et al.* Roles of meteorological conditions in COVID-19 transmission on a worldwide scale. *medRxiv* **11**, 2020.03.16.20037168. 10.1101/2020.03.16.20037168 (2020).

[12] Oliveiros, B., Caramelo, L., Ferreira, N. & Caramelo, F. Role of temperature and humidity in the modulation of the doubling time of COVID-19 cases. *medRxiv*. 10.1101/2020.03.05.20031872 (2020).

[13] Zhu Y, Xie J, Huang F, Cao L (2020). Association between short-term exposure to air pollution and COVID-19 infection: Evidence from China. Sci. Total Environ..

[14] Fernández Richard J (2013). La administración del Estado y las municipalidades en Chile. Rev. IUS.

[15] Luo Y (2013). Lagged effect of diurnal temperature range on mortality in a subtropical megacity of China. PLoS ONE.

[16] Pinheiro, S. de L. L. de, Saldiva, P. H. N., Schwartz, J. & Zanobetti, A. Isolated and synergistic effects of PM10 and average temperature on cardiovascular and respiratory mortality. *Rev. Saude Publ.* **48**, 881–888 (2014). 10.1590/S0034-8910.2014048005218 PMC4285836 26039390

[17] Ficetola, G. F. & Rubolini, D. Climate affects global patterns of COVID-19 early outbreak dynamics. *medRxiv* 2020.03.23.20040501. 10.1101/2020.03.23.20040501 (2020).

[18] Xie J, Zhu Y (2020). Association between ambient temperature and COVID-19 infection in 122 cities from China. Sci. Total Environ..

[19] Tan J (2005). An initial investigation of the association between the SARS outbreak and weather: with the view of the environmental temperature and its variation. J. Epidemiol. Commun. Health.

[20] Pani SK, Lin N-H, RavindraBabu S (2020). Association of COVID-19 pandemic with meteorological parameters over Singapore. Sci. Total Environ..

[21] Zhu Y, Xie J, Huang F, Cao L (2020). The mediating effect of air quality on the association between human mobility and COVID-19 infection in China. Environ. Res..

[22] Sanchez-Lorenzo A (2021). Did anomalous atmospheric circulation favor the spread of COVID-19 in Europe?. Environ. Res.

[23] Yan, W. *et al.* Atmospheric pressure and population density as super-factors influencing the transmission of coronavirus disease 2019 (COVID-19). 10.21203/rs.3.rs-93707/v1 (2020).

[24] Lolli S, Chen YC, Wang SH (2020). Impact of meteorological conditions and air pollution on COVID-19 pandemic transmission in Italy. Sci. Rep..

[25] Cambaza EM, Viegas GC, Cambaza CM (2020). Potential impact of temperature and atmospheric pressure on the number of cases of COVID-19 in Mozambique, Southern Africa. JPHE.

[26] Lin S (2020). Region-specific air pollutants and meteorological parameters influence COVID-19: A study from mainland China. Ecotoxicol. Environ. Saf..

[27] Yuan J (2006). A climatologic investigation of the SARS-CoV outbreak in Beijing, China. Am. J. Infect. Control.

[28] Chan, K. H. *et al.* The effects of temperature and relative humidity on the viability of the SARS coronavirus. *Adv. Virol.* **2011** (2011). 10.1155/2011/734690 PMC3265313 22312351

[29] Wang J, Tang K, Feng K, Lv W (2020). High temperature and high humidity reduce the transmission of COVID-19. SSRN Electron. J..

[30] Metz JA, Finn A (2015). Influenza and humidity—Why a bit more damp may be good for you!. J. Infect..

[31] Lowen AC, Mubareka S, Steel J, Palese P (2007). Influenza virus transmission is dependent on relative humidity and temperature. PLoS Pathog.

[32] Yang, W. & Marr, L. C. Dynamics of airborne influenza A viruses indoors and dependence on humidity. *PLoS One* **6** (2011). 10.1371/journal.pone.0021481 PMC3123350 21731764

[33] De Jong, J. C., Trouwborst, T. & Winkler, K. C. Mechanisms of inactivation of viruses and macromolecules in air. *Airborne Transm. Airborne Infect.* 124–130 (1973).

[34] Moriyama M, Hugentobler WJ, Iwasaki A (2020). Seasonality of respiratory viral infections. Annu. Rev. Virol..

[35] Bukhari, Q. & Jameel, Y. Will coronavirus pandemic diminish by summer? *SSRN Electron. J.*10.2139/ssrn.3556998 (2020) .

[36] Sarricolea P, Herrera-Ossandon M, Meseguer-Ruiz Ó (2017). Climatic regionalisation of continental Chile. J. Maps.

[37] INE. *Síntesis de Resultados Censo 2017*. https://www.censo2017.cl/descargas/home/sintesis-de-resultados-censo2017.pdf (2018).

[38] INE. *Estimaciones y Proyecciones de la Población de Chile 2002–2035. Totales Regionales, Población Urbana y Rural. Síntesis de Resultados Instituto Nacional de Estadísticas Junio 2019* (2019).

[39] DGA. *Atlas del Agua Chile 2016* (2016).

[40] INE. *Adultos Mayores en Chile: ¿Cuántos Hay? ¿Dónde Viven? ¿Y en qué Trabajan?* https://www.ine.cl/prensa/detalle-prensa/2020/04/15/adultos-mayores-en-chile-cuántos-hay-dónde-viven-y-en-qué-trabajan (2020).

[41] MINSAL. *Informe Epidemiológico Enfermedad por SARS-Cov-2 (COVID-19) Chile 17–08–2020*. https://www.minsal.cl/wp-content/uploads/2020/08/Informe-epidemiologico-43-MINSAL.pdf.

[42] INIA. *Agrometereología Red Agrometereológica de Inia*. https://agrometeorologia.cl/ (2020).

[43] Yahia, E. M. Introduction. In *Postharvest Technology of Perishable Horticultural Commodities* 1–41. 10.1016/B978-0-12-813276-0.00001-8 (Elsevier, 2019).

[44] SINCA-MMA. *SINCA Sistema de Información Nacional de Calidad del Aire*. https://sinca.mma.gob.cl/index.php/ (2020).

[45] CONAF. *Proyecto Catastro y Evaluación de los Recursos Vegetacionales Nativos de Chile*. http://sit.conaf.cl/ (2013).

[46] IDE Chile. *IDE Chile Infraestructura de Datos Geoespaciales*. http://www.ide.cl/ (2020).

[47] Marquardt DW (1970). Generalized inverses, ridge regression, biased linear estimation, and nonlinear estimation. Technometrics.

[48] Chen, T. & Guestrin, C. XGBoost: A scalable tree boosting system. *Proc. ACM SIGKDD Int. Conf. Knowl. Discov. Data Min.* **13**–**17**, 785–794 (2016).

[49] Mousavi SS, Schukat M, Howley E (2018). Deep reinforcement learning: An overview. Lect. Notes Netw. Syst..

[50] R Core Team. *R: A Language and Environment for Statistical Computing*. https://www.R-project.org. (R Foundation for Statistical Computing v. 3.2.5, 2018).

